# Supplementary material for: Are carbon emissions trading and green financial instruments synergistic? -Comprehensive quantitative research based on content analysis
Source: PLoS One. 2024 Mar 7;19(3):e0298601. doi: 10.1371/journal.pone.0298601 (PMC10919601; doi:10.1371/journal.pone.0298601)
Supplement: S1 Data — (ZIP) [file pone.0298601.s001.zip › Data/地级市Z_F工作报告.zip!/╝¬┴╓/░╫│╟╩╨/└┤╫╘╣1⁄2╓┌║┼í╛┬φ┐╦╩2╛▌═°í┐_2005.docx]

各位代表：

　　我代表白城市人民政府，向大会报告政府工作，请予审议，并请市政协各位委员提出意见。

　　一、2004年工作回顾

　　2004年，在市委的正确领导下，在市人大依法监督和市政协民主监督下，市政府坚持以经济建设为中心，团结带领全市人民在振兴东北老工业基地的开局之年，抢抓机遇，开拓进取，努力克服严重旱灾和各种困难，各项事业都取得了新成绩，完成了市三届人大一次会议确定的政府各项任务。

　　——国民经济实现较快增长。预计全年地区生产总值完成137.5亿元，同比增长13.2%；固定资产投资53亿元，同比增长10.3%；全口径财政收入95812万元（扣除农业税），同比增长16%；城镇居民人均可支配收入7396元，同比增长15.1%；农民人均纯收入在大灾之年达到2050元。

　　立足培育壮大优势产业，加大增产增效力度。全市规模以上工业完成产值54亿元，同比增长16.5%；工业增加值完成18.5亿元，同比增长16%；实现利润2.1亿元，同比增长11%。开工技改项目24项，开发新产品165种。民营经济发展步伐加快，实现产值89.3亿元，同比增长22%。

　　全力组织农村各项工作，农业在大灾之年取得可喜成果。面对百年一遇的严重旱灾，集中人力、物力、财力抗灾自救，努力减少灾害造成的损失。推进农业园区建设，新建万元田棚、股份牧业等园区53个。抓好“一免三补”政策落实，充分调动农民种粮积极性。加强生态建设，完成水利综合工程量970万立方米，造林43.5万亩，瀚海桑田工程取得了明显成效。

　　大力发展商贸经济，内外贸易取得新进展。社会消费品零售总额完成65.6亿元，同比增长16%。新建白城经济开发区蔬菜副食品综合市场、洮北区农机市场等5处大型市场，经营规模进一步扩大，市场功能不断完善。积极发展对外贸易，海关统计外贸进出口额2414万美元，其中出口2011万美元，同比增长47.3%。

　　——改革开放步伐加快。坚持以体制和机制创新为重点，通过转换职工身份、出售产权、国有股权减持、破产退出等途径，推进了国有企业改革。白城尼特固公司、大安云鹤汽车座椅总成公司、白城百琦药业等一批企业通过重组整合焕发了生机和活力。有序推进了完善城镇社会保障体系试点、粮食流通体制改革、免征农业税改革试点工作。科技、教育、文化、卫生等方面改革取得了新进展。

　　坚持企业为主与政府推动相结合，开展了赴德国、日本、韩国、香港等国家和地区的经贸洽谈活动，积极参加港洽会、西洽会、重庆和成都投资贸易会等招商引资活动，举办各种形式的项目推介会，积极拓展招商引资渠道，全市招商引资到位资金45亿元。开发区建设、生态旅游开发、劳务输出等工作取得了新成绩。

　　——项目建设全面推进。抓住振兴东北老工业基地战略机遇，积极推进“项目开发建设年”各项工作，项目建设领域不断拓宽，投资渠道日益多元化。建设千万元以上重点项目126项，完成投资22亿元。开工建设了白城派克公司汽车原线及出口线束、镇赉格法玻璃改造、洮南风力发电塔筒制造、通榆葵花园区、大安永兴米业加工、洮北铝塑门窗型材等一批项目。启动了洮南、洮北各5万千瓦风电项目，通榆团结风电场40万千瓦特许经营权项目已经国家发改委批复并完成了前期工作。

　　——基础设施建设迈出新步伐。继续实施了城市开发建设管理总体战，完成投资37.3亿元。全市新建、改造、维修、硬化道路271条，新增绿地153.5万平方米。新建住宅110万平方米，住房公积金工作全面启动。亮化、蓝天等工程取得成效，土地市场秩序进一步规范。城市卫生状况、交通秩序和市容市貌有了新改观。加快公路建设，科铁线二级公路完成64公里，村村通等级公路完成1763公里。信息和通讯设施进一步完善。

　　——各项社会事业持续发展。积极推进科教兴市战略，实施各级科技发展项目150项。加快教育结构调整，白城卫校升格为白城医学高等专科学校，恢复设置了白城教育学院，基础教育水平不断提高。文化体育事业蓬勃发展，举办了“纪念白城设地50周年”等大型文艺活动，广播电视节目获省级一等奖17项；我市运动员在国际马拉松男子10公里比赛中获金、银牌各1枚，在全省各类比赛中获金牌41枚。加强了公共卫生体系建设和疾病预防控制工作，禽流感防治和非典防控等取得成效。计划生育工作全面完成了省下达计划。《白城年鉴》获全国首届年鉴质量综合一等奖。完成了第一次经济普查的准备工作。民族、宗教、侨务、老龄、妇女儿童、残联、红十字会等事业有了新发展，国防后备力量建设、双拥、人防、防震减灾等工作取得了新成绩。

　　——城乡社会保持稳定。积极落实就业和再就业政策，开发就业岗位5万个，新增就业4.1万人。加强了养老统筹、失业、医疗保险基金征缴、扩面和发放工作，全市养老保险覆盖率达97%，省级、集体统筹养老金和基本医疗保险、失业保险基金发放均达100%。抓好农村救灾救济和城市低保工作，开展了社会捐助和互助互济活动，符合条件的低保对象全部纳入低保范围。推进社区建设，建立各级“阳光超市”和社区连锁店45个。进一步落实信访领导责任制，努力化解和妥善处理各种社会矛盾。强化整治和监管，确保了生产、消防、交通安全。严厉打击各种严重刑事犯罪和“法轮功”等邪教组织，加强社会治安综合治理，保持了社会稳定。

　　——民主法制和政府自身建设得到加强。认真执行人大决议和决定，定期向人大常委会报告工作，积极支持政协和各民主党派、工商联参政议政，自觉接受人大依法监督和政协民主监督。承办人大代表建议61件、政协委员提案和建议案49件，全部办理完毕。努力改善发展软环境，组织力量对行政许可项目进行了审核清理，保留233项，取消82项。加大了法制宣传教育和依法治市工作力度，强化人民调解工作。组织开展了行政执法检查，做好行政复议工作，促进依法行政。充分发挥市长和县（市、区）长公开电话作用，群众投诉办结率和满意率分别达到97.2%和95.3%。有效开展了经济监督和领导干部经济责任审计，我市被授予领导干部经济责任审计全国先进地区称号。认真落实廉洁自律各项规定和“一岗双责”责任制，纠正行业和部门不正之风，加大案件查办力度，廉政建设取得了新进展。

　　2004年所取得的成绩，是全市各级干部和广大人民群众齐心协力、拼搏奋斗的结果；是社会各界、各方面大力支持的结果。在此，我代表市政府向全市工人、农民、知识分子、各阶层群众和各级干部，向各级人大、政协、民主党派、工商联、人民团体及各界人士，向驻白部队、武警官兵、公安司法干警和中省直驻白单位，向对我们工作给予关心和帮助的离退休老领导、老同志表示衷心的感谢和崇高的敬意！

　　同时，我们也清醒地看到全市经济社会发展中存在的问题，主要是：经济总量小，结构不优，财力较弱，缺少大项目支撑和拉动；民营经济规模不大，实力不强；由于农村遭受严重旱灾和城镇就业再就业形势严峻，城乡居民的生产生活面临许多实际困难；社会不安定因素仍然存在；经济发展环境需要进一步改善等等。对这些问题，我们要高度重视，采取更加有力着实的措施认真加以解决。

　　二、2005年经济社会发展目标、任务

　　2005年是推进白城加快发展至关重要的一年。市政府工作总体要求是：以邓小平理论和“三个代表”重要思想为指导，全面贯彻落实党的十六届四中全会、省委八届六次全会和市委三届八次全会精神，紧紧抓住振兴东北老工业基地的战略机遇，立足全面提速，加快发展步伐，加快改革开放，加快项目建设，加快招商引资，全面优化投资环境，大力推进工业化、城镇化和农业产业化，切实加强精神文明和民主法制建设，努力实现全市经济超常发展和社会全面进步。

　　主要预期目标：地区生产总值158亿元，同比增长15%；固定资产投资76亿元，同比增长43.5%；全口径财政收入10.2亿元，按可比口径同比增长13%；城镇居民人均可支配收入8060元，同比增长9%；农民人均纯收入2750元，同比增长34%。

　　做好今年政府工作，必须坚持把加快发展作为第一要务，以时不我待的紧迫感、奋力赶超的责任感，全力抓发展，强力抓落实，形成推动工作的强劲态势，使发展由快走变为快跑。必须坚持抓重点促全局，突出项目建设，突出招商引资，突出全民创业，动员一切力量，抓住一切机遇，用足一切政策，挖掘一切潜力，合力推动白城更快更好地发展。必须坚持把实现人民群众的根本利益作为工作的出发点，千方百计提高人民群众生活水平，使全市人民普遍共享加快发展的成果。

　　为了完成全年目标，主要抓好以下十项工作：

　　（一）实施“百项工程”攻坚，全力抓好项目建设，加快扩充经济总量

　　项目建设是白城经济发展的关键，要把项目建设作为经济工作的重中之重，全市动员，全民参与，全面推进。

　　要突出抓好100项千万元以上重点项目，投资45亿元。组织好白城金鹏公司3万台重型汽车变速箱总成、纺织公司8.5万锭纺纱、吉林马应龙药业200万支盐酸关附甲素注射液、大安3000吨玉米黄色素及大豆蛋白、洮南百万亩山杏、镇赉7万吨有机黑大豆深加工、通榆120座风机塔筒、洮北区20万立方米承重砌块等项目建设。坚持新开项目抓进度，续建项目抓竣工，建成项目抓达产，确保项目上水平。实行“百项工程”包保责任制，落实项目责任单位和责任人，层层签订责任状，定期调度，经常检查，逐级考核，奖罚分明，奖惩兑现，坚决完成“百项工程”攻坚任务。

　　积极主动争取项目，增强项目建设后续能力。紧紧抓住振兴东北老工业基地的战略机遇，把握国家产业政策，做好霍白铁路、白城第二发电厂、白城热电厂扩建、引嫩入白工程、风电和石油开发等大项目的争取工作。积极申报国债项目，认真完善待批项目，精心挑选拟报项目，滚动培育备选项目。努力做好前期工作，提高项目申报质量，力争取得更大成效。

　　培育项目投资主体，提升项目市场化运作水平。要通过招商引资、国企改制、全民创业，积极发展合格的项目投资主体，培育从事项目谋划运作的各类中介机构，在项目策划、市场分析、技术咨询、设计包装以及资本运作等方面形成专业化、市场化的运作体系，由市场和企业承担更多的项目运作职能，提高项目谋划和建设水平。同时，要搞好“十一五”规划的编制工作，全面谋划好白城未来的发展。

　　（二）以工业园区为载体，以六大产业为核心，加快壮大工业实力

　　工业是立市之本、富市之源。坚持探索走新型工业化道路的有效途径，转变增长方式，扩大产业规模。规模以上工业增加值同比增长20%。

　　抓好工业园区建设，全力打造加快白城发展的“希望工程”。坚持高起点规划，从市场预测、产业结构、自然资源和社会条件等多方面进行综合评价，合理确定园区功能、布局和主导产业发展方向。抓好“七通一平”和公共设施建设，落实各项优惠政策和扶持措施，以最优的政策、最快的审批、最低的收费、最好的服务，吸引投资者到园区开发兴业，形成规模和集群发展优势。

　　培育重点产业，扩大工业规模。能源工业重点抓好风电、火电和石油开发及配套设备生产，做大做强能源产业，提高工业经济的竞争优势；汽车零部件工业在为大企业配套生产的同时，积极寻求并购重组，走合资合作、加快发展之路；纺织服装工业加快设备更新改造和技术创新，实现生产规模的快速扩张；医药工业在完成GMP改造的基础上，抓好生物药、植化药、合成药的科技开发，提高产品的科技含量；农产品加工业要搞好特色农畜产品深加工，延长加工链条，多出终端产品，扩大经营规模；建材工业立足资源开发、新型材料推广应用，进一步提高生产能力。努力推进上市融资工作，早日实现由产品经营向资本经营的跨越。

　　组织好增产增效工作，提高工业运行质量。加强经济监测分析，千方百计挖掘潜力，确保完成增产增效目标。着力推进技术创新，加快技改项目建设，抓好新产品和高新技术产品开发，引导企业积极开拓国内外市场，提高产品市场占有率。

　　（三）立足农业增效和农民增收，大力发展适应农业和效益农业，加快发展农村经济

　　统筹城乡发展，积极建立农民增收的长效机制，推进农业产业化。农业产值同比增长15%。

　　突出特色，推进结构调整。认真落实“一免三补”政策，稳步提高粮食综合生产能力，扩大绿豆、葵花、蓖麻、红干椒、棉花等特色农产品的种植面积，做大杂粮杂豆产业。积极引进优良

　　品种，开发无公害绿色有机农产品，提高市场竞争力。大力发展畜牧业，重点发展奶牛、肉牛和白鹅业，逐步把畜牧业培育成农村主导产业，扩大规模提高质量。

　　培育优势，推进产业化经营。大力发展万元田棚、园区农业、特色农业和瀚海桑田工程。抓好农产品基地建设和加工业，促进专业化、集约化生产。培育壮大龙头企业，发挥带动作用。积极发展农村各类经济合作组织，提高农民市场化组织程度。加强农业信息服务和市场开发，提高农业经济效益。

　　标本兼治，推进生态建设。立足改善农业生产条件和生态环境，抓好水利建设，组织好江河堤防治理、水库除险加固、节水灌溉、引水蓄水等工程建设，进一步增强过境江河水、洪水蓄滞利用能力和人工增雨能力。加快造林绿化，完成造林30万亩，营造生态草70万亩，瀚海桑田工程完成植桑7.5万亩，养蚕5000 张。强化土地资源管理、保护耕地和芦苇开发工作。注重生态环保和资源的可持续利用，培育发展循环经济。

　　拓展渠道，推进劳务输出。加强组织，搞好培训，完善服务，建立基地，加快农村劳动力转移，推动我市农民走出土地、走出农村、走出白城闯世界，提高非农收入水平。全年劳务输出30万人（次）。

　　（四）充分发挥三省交界的区位优势，以现代服务业和旅游业为重点，加快发展第三产业

　　立足增强辐射带动功能，全面搞活服务业，积极发展旅游业，培育和完善市场体系。社会消费品零售总额同比增长12%。

　　积极发展信息、中介、会计、审计、律师等现代服务业，推进连锁经营、物流配送等新型流通业态，不断提高商贸、餐饮、仓储、交通运输等传统服务业水平。进一步增强金融服务功能，加大对优势产业和重点项目的贷款投放力度，促进银企合作与共赢。

　　加快发展有特色、成规模、辐射力强的新型市场，巩固完善杂粮杂豆、禽畜瓜椒等专业市场。积极抓好人才、劳动力、技术、信息、房地产等各类要素市场。强化市场管理，坚决打击制售假冒伪劣等行为，维护经营者和消费者利益。

　　大力开发品牌旅游。立足草原风光、湿地生态、地域文化、风电景观等特色旅游资源，进一步完善旅游发展规划。坚持投资主体多元化、经营市场化，加强向海、莫莫格、查干浩特和月亮湖等景区景点建设，开发特色旅游产品，培育旅游品牌，逐步融入国内外旅游大市场。

　　（五）坚持以国企改革为重点，积极推进各项改革，加快体制和机制创新

　　要集中力量打一场国企改革攻坚战，按照“改制范围放开、改制形式放开、出让对象放开”的原则，从企业实际出发，坚持“一企一策”，年底前基本完成国有企业改制任务。千方百计筹措改革成本，充分用好用足上级政策，采取产权出售、招商重组、引资改造、同业整合、管理层收购，以及兼并、破产等多种形式，全力推进改革，确保整体改制到位、债权债务处理到位、职工身份转换到位和国有资本退出到位。完善国有资产监管体制，防止国有资产流失，保护好职工合法权益。

　　抓好完善城镇社会保障体系试点工作，及时研究解决试点中出现的新情况、新问题，积极工作，加快步伐。推进粮食流通体制改革，确保年底前基本完成国有粮食购销企业改革任务。组织好免征农业税改革试点工作，落实各项政策，完善配套措施。继续抓好科技、教育、文化、卫生等方面改革，增强发展动力。

　　（六）推动全民创业，着力壮大民营经济，加快培育新的经济增长点

　　要把发展民营经济作为焕发经济活力的一项战略性措施，上争下促，外引内培，推动民营经济形成强劲发展的新优势。全年实现增加值、税收分别增长24%和20%。

　　坚持多措并举，增加民营企业数量。围绕全民创业，鼓励本地能人兴办一批，推进国企改制整合一批，扶持下岗职工合办一批，支持机关事业单位人员离岗领办一批，通过招商引资引进一批。个体工商户和私营企业发展到10.6万户，从业人员达到18.8万人。扶持和帮助有条件的民营企业上规模、上水平。

　　强化政策扶持，放宽市场准入条件，扩大准入领域，凡国家法律法规未予限制的领域都要向民营经济放开，凡符合国家产业政策的都要放手让民营企业发展。拓宽民营企业融资渠道，积极搞好信息、技术、智力、咨询等服务，推动民营经济实现大发展。

　　（七）全方位开展招商引资，扩大合资合作领域，加快对内对外开放步伐

　　充分利用国际国内两个市场、两种资源，积极引进项目、资金和战略合作伙伴，借助外力发展壮大。

　　加大招商引资力度，提高融资水平。坚持企业为主、政府推动、全民参与，创新招商引资机制。积极参加“广交会”、“厦洽会”、“深圳高交会”等经贸洽谈和招商引资活动，组织好赴苏州、无锡、宁波等地开展转移产业承接招商。千方百计引进战略投资伙伴，吸引境外大企业、大集团到白城投资兴业。采取敲门招商、以商招商、中介招商、网上招商等形式，不断扩大招商引资成果。全市招商引资完成49亿元。

　　拓展国际市场，扩大出口规模。在巩固原有出口市场的同时，着力发展新的贸易伙伴。培育具有国际竞争力的产业，开发本地优势强、市场容量大、出口前景好的产品。抓好出口基地建设，扩大出口能力。引导企业通过多种途径建立国外销售网络，鼓励有实力的企业到国外办厂设店，带动和促进全市产品出口。海关统计进出口额同比增长11.8%

　　推进开发区建设，培育发展优势。充分发挥开发区示范带动作用，创新管理体制和运行机制，加强基础设施建设，优化投资环境，增强招商引资和项目建设的吸引力。

　　（八）抓好基础设施建设，强化管理和维护，加快改善城乡面貌

　　运用市场机制经营城市，继续开展城市开发建设管理总体战。全市计划投资32.9亿元，其中市区投资18.6亿元，各县（市）投资14.3亿元。市区集中抓好与百姓生活密切相关的九项民利工程（132件实事），主要是以污水处理续建、给水供热管网、生活和医疗垃圾无害化处理等为重点的基础设施建设工程，以淮河路、洮儿河路、阳光街等新建和人行道铺装为重点的道路建设工程，以游园、街路绿化、环卫设施完善等为重点的环境建设工程，以玫瑰园、阳光嘉园、鹤林苑等为重点的住宅建设工程。各县（市）要按计划完成各项开发建设任务，努力提升城乡环境质量。

　　加强城市管理，巩固开发建设成果。加大综合执法力度，创新城市管理机制，打击破坏市政公用设施行为。从改善卫生、美化环境、整顿秩序入手，综合整治城市环境，切实解决脏乱差问题，努力提高城市管理水平。

　　立足推进城镇化，加快小城镇建设。充分发挥资源优势，发展特色经济，培育主导产业，用工业化带动城镇化。今年培育1-2个示范镇。

　　同时，抓好公路建设，科铁线二级公路要全部竣工，村村通等级公路确保完成1000公里。

　　（九）全面实施科教兴市战略，积极发展各项社会事业，加快推进社会全面进步

　　深入贯彻落实《公民道德建设实施纲要》，广泛开展群众性精神文明创建活动，提高城乡文明程度。积极推进科技创新，努力争取和组织实施国家、省科技发展项目，抓好科技示范园区建设，培育和发展高新技术企业、民营科技企业。加强大专院校、科研机构、企业的联合协作，促进科技成果转化。积极发展高中教育和职业教育，努力办好高等教育，组织好“控辍”和对口支援农村薄弱校工作，提高义务教育水平。发展文化体育事业，强化社区文化、企业文化和乡村文化建设，推进竞技体育和群众体育协调发展。完善疾病预防控制体系和医疗救治体系，提高医疗服务水平。抓好计划生育工作，确保完成全年人口计划。充分发挥广播电视宣传作用，营造良好的舆论氛围。进一步加强国防后备力量建设，开展好双拥共建活动。认真做好第一次经济普查工作。积极做好民族、宗教、侨务、地方志、防震减灾、气象、人工降雨、人防、老龄、妇女儿童、残联、红十字会等工作，加快社会事业发展步伐。

　　（十）切实做好关系群众利益的各项工作，努力化解各种矛盾，营造加快发展的和谐稳定社会环境

　　坚持把推进全民创业、扩大就业再就业作为实现和维护群众利益的首要任务，进一步落实就业政策，深入开展“创业促就业”活动，多渠道开发就业岗位，组织好培训，提高群众的就业能力。以完善养老、失业、医疗保险制度为重点，不断扩大社会保障覆盖面。推进农村扶贫开发和城市扶困，尽快使城乡贫困人口脱贫。抓好灾区救济工作，解决好灾民的口粮、蔬菜、烧柴、饮用水和生产等方面的问题，确保灾区群众生活。继续做好住房公积金和新型农村合作医疗试点等工作。严格落实信访责任制和责任追究制，加大矛盾纠纷排查调处力度，努力化解社会矛盾。抓好市长和县（市、区）长公开电话工作，切实为民排忧解难。加强社会治安综合治理，严厉打击各种刑事犯罪和经济犯罪，积极推进社会治安防控体系建设。继续开展同“法轮功”及其它邪教组织的斗争，有效防范和惩治各种非法活动。全力抓好安全生产，坚决遏制重大安全事故的发生。

　　三、加强政府建设，提高行政效能

　　加快白城发展必须加强政府建设。我们要坚持科学的发展观，树立正确的政绩观，认真履行好经济调节、市场监管、社会管理和公共服务职能，努力建设为民造福的服务政府、务实高效的责任政府。

　　进一步解放思想。解放思想是加快发展的前提。我们要围绕发展强化五个意识。一是强化加快发展意识。发展是第一要务，发展是人民群众最迫切的愿望，发展也是解决白城一切问题的前提和基础。要突破常规思维、突破常量安排、突破常态运行，尽快形成全心全意求发展、千方百计谋发展、全力以赴促发展的局面。二是强化重点发展意识。突出项目建设这个重点，努力在全市上下形成“以经济建设为中心、以项目建设为重、以工业项目为主”的共识，用推进项目建设的成效来评价干部、衡量工作。各级干部要“言必谈项目，行必促发展” 。三是强化科学发展意识。按经济规律办事，既不能超越现实去做做不了的事情，更不能逃避历史责任不去做经过努力能够做到的事情，坚定信念，充满信心，奋力拼搏。四是强化自主发展意识。省里提出要支持白城发展，这是我们面临的难得机遇，但不能坐等，积极争取特殊政策，用足用活“普惠”政策。发挥群众的主观能动性、创造性，自强不息，全民创业，齐心合力促发展。五是强化发展能力意识。适应新的形势和任务，充分发挥各级领导机构和领导干部的执政行为主体作用，与时俱进，开拓创新，提高加快发展、协调发展、稳定发展的能力。

　　进一步加强政风建设。坚持把加快发展的立足点放到真抓实干上，把求真务实的精神体现到政府各项具体工作中，重实干、求实效，坚决反对形式主义、急功近利，不允许任何部门、任何人搞任何形式的弄虚作假；不允许任何部门、任何人以任何借口不作为。要在机关中开展好“四服务”活动，真正服务企业、服务项目、服务投资者、服务群众。切实转变工作方式，加快工作节奏，多深入基层、多开展现场办公、多走出去跑项目搞招商、多为投资者和群众解决实际问题；减少会议、减少文件、减少事务性活动、减少领导干部公务活动的陪同人员，集中精力抓大事、抓工作、抓落实。积极稳妥地做好政府部门机构改革工作，理顺职能，提高行政效能。建设高素质的公务员队伍，使这支队伍中更多的人都能对事业充满激情，对群众充满感情，扎实工作，奋力作为。

　　进一步加强民主法制建设。自觉接受人大的依法监督和政协的民主监督，完善向人大报告工作和向政协通报工作制度，认真办理人大代表建议和政协委员提案，密切同各民主党派、工商联和人民团体的联系。建立健全社情民意反映制度、社会听证制度和专家咨询制度，推进决策科学化、民主化。扎实开展法制宣传教育，提高公民的法律素质。加强依法治市，积极开展法律服务。完善政府法制工作，强化执法监督，提高依法行政水平。

　　进一步加强软环境建设。认真贯彻《行政许可法》，对非许可审批项目全面进行清理，凡是能够精减的审批一律精减，凡是能够取消的收费必须取消。大力实施“阳光政务”，继续推进政务公开。加强执法队伍建设，规范行政执法行为。强化政务大厅的职责和功能，着力解决好“体外循环”问题。抓好各项政策措施落实，加强监督检查，严肃查处“涉软”案件，对损害创业者利益、侵害投资者权益的，不管是谁，发现一个处理一个，为各类市场主体创造规范的政务环境、公平的法制环境、文明的社会环境、诚信的市场环境。

　　进一步加强廉政建设。认真落实党风廉政建设责任制，严格执行领导干部廉洁自律各项规定，努力从源头上预防和治理腐败。以解决群众反映的突出问题为重点，坚决纠正损害群众利益的部门不端行为和行业不正之风。积极开展执法监察，认真抓好以预算执行和专项资金审计为重点的经济监督和领导干部经济责任审计，以反腐倡廉的实际成果取信于民，不孚众望。

　　各位代表，白城的发展需要我们不懈的努力，白城的美好未来要靠我们共同来创造，使命光荣，责任重大。我们要在省委、省政府和市委的领导下，在市人大、市政协的支持监督下，凝聚全市人民的智慧和力量，恪尽职守，拼搏奋斗，以坚韧不拔的毅力、百折不挠的精神、脚踏实地的作风，齐心创造各项工作的新业绩，携手谱写白城发展的新篇章！
